# Supplementary figures and images for: Cell cycle-dependent gene networks for cell proliferation activated by nuclear CK2α complexes
Source: Life Sci Alliance. 2023 Oct 31;7(1):e202302077. doi: 10.26508/lsa.202302077 (PMC10618106; doi:10.26508/lsa.202302077)

Figure 3A Preparation of CK2 $\alpha$ -ChIP followed by western blotting with anti-CK2 $\alpha$  antibody.

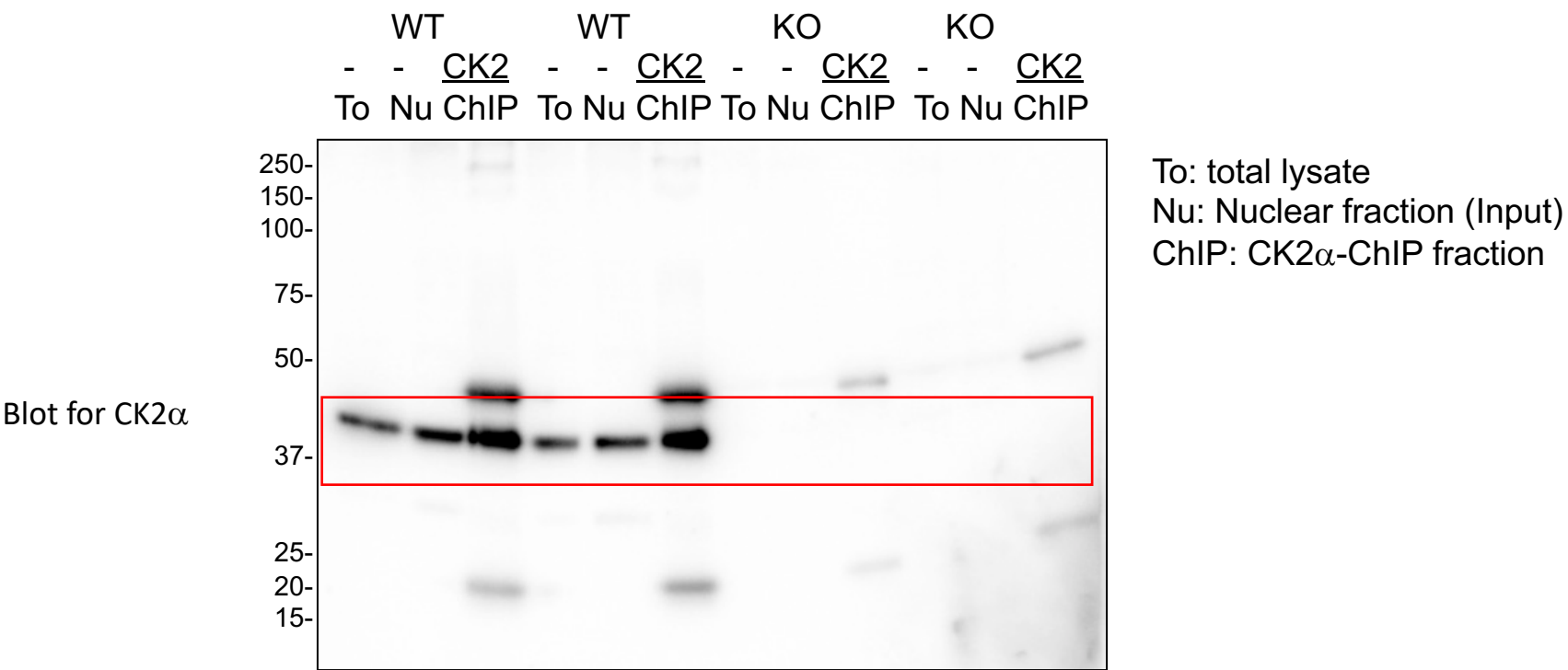

Supplement: Supplementary file 5 [file LSA-2023-02077_SdataF3.pdf]
