# Supplementary material for: Cell cycle-dependent gene networks for cell proliferation activated by nuclear CK2α complexes
Source: Life Sci Alliance. 2023 Oct 31;7(1):e202302077. doi: 10.26508/lsa.202302077 (PMC10618106; doi:10.26508/lsa.202302077)
Supplement: Supplementary file 8 [file LSA-2023-02077_SdataFS3.2.zip › Figure 3SC_HommaMK.pptx]

## Slide 1
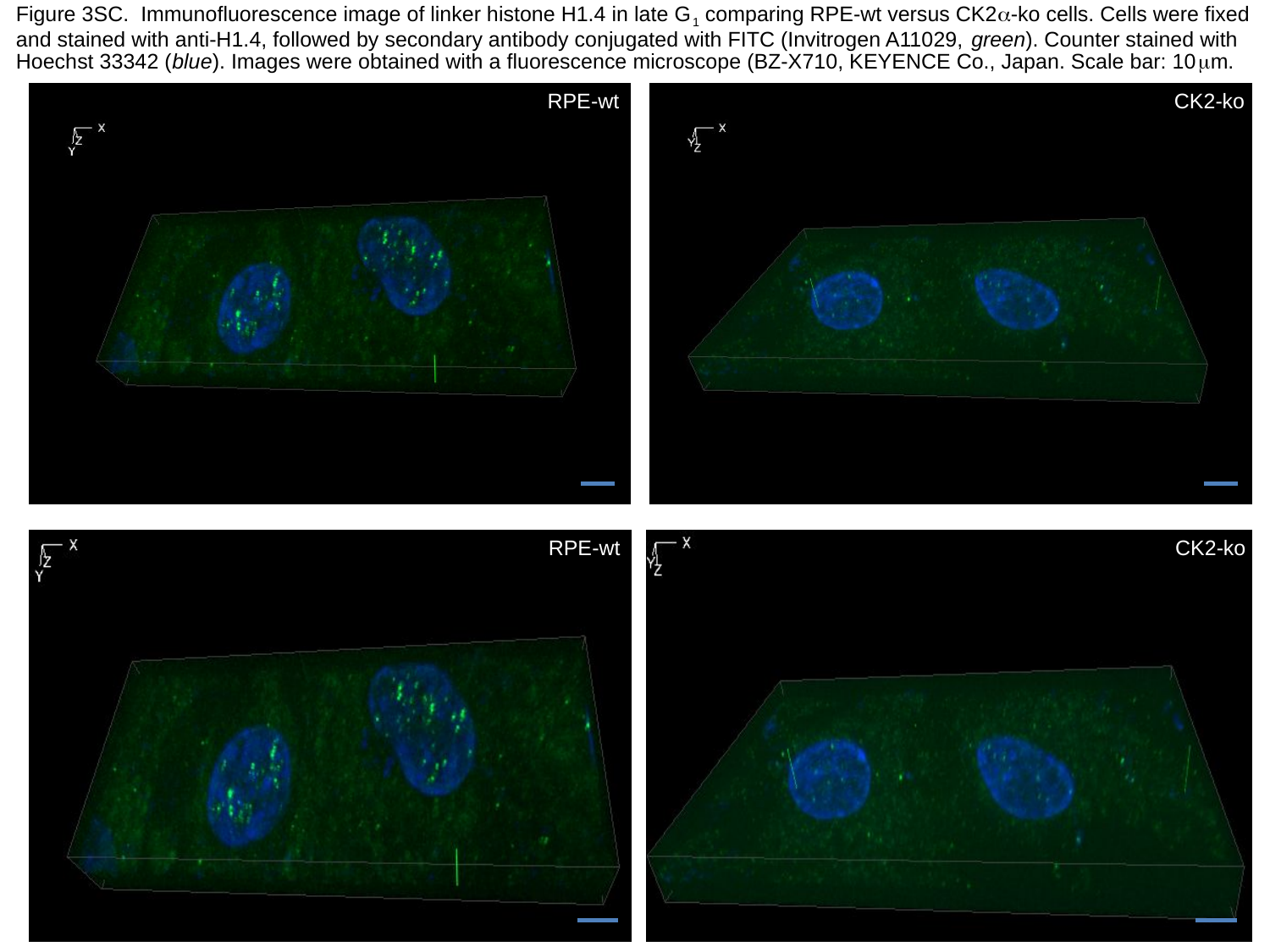

Figure 3SC. Immunofluorescence image of linker histone H1.4 in late G1 comparing RPE-wt versus CK2a-ko cells. Cells were fixed
and stained with anti-H1.4, followed by secondary antibody conjugated with FITC (Invitrogen A11029, green). Counter stained with
Hoechst 33342 (blue). Images were obtained with a fluorescence microscope (BZ-X710, KEYENCE Co., Japan. Scale bar: 10mm.
RPE-wt CK2-ko
RPE-wt CK2-ko
